# Case report: Rescue treatment with add-on selexipag in a preterm infant with suprasystemic pulmonary hypertension, pulmonary capillary hemangiomatosis, and isolated pulmonary vein stenosis

**DOI:** 10.3389/fcvm.2022.984698

**Published:** 2022-12-09

**Authors:** Hosan Hasan, Klea Hysko, Thomas Jack, Jens Dingemann, Martin Wetzke, Georg Hansmann

**Affiliations:** ^1^Department of Pediatric Cardiology and Critical Care, Hannover Medical School, Hannover, Germany; ^2^European Pediatric Pulmonary Vascular Disease Network, Berlin, Germany; ^3^Department of Pediatric Surgery, Hannover Medical School, Hannover, Germany; ^4^Department of Pediatric Pulmonology, Hannover Medical School, Hannover, Germany

**Keywords:** bronchopulmonary dysplasia (BPD), pulmonary hypertension (PH), neonatology, selexipag, prematurity, intensive care

## Abstract

An extremely dystrophic, premature female infant, born at 25 3/7 weeks of gestational age (birth weight: 430 g) with severe pulmonary hypertension (PH), was admitted to our neonatal intensive care unit (ICU) requiring cardiorespiratory support, including mechanical ventilation and pulmonary vasodilators such as inhaled nitric oxide (iNO) and continuous intravenous sildenafil infusions. The diagnosis of bronchopulmonary dysplasia (BPD) was made. A hemodynamically relevant, persistent ductus arteriosus (PDA) was surgically ligated after failed pharmacologic PDA closure using indomethacin and ibuprofen. The patient was discharged with an estimated 2/3 systemic pulmonary artery pressure. One month after hospital discharge, on low-flow oxygen supplementation (0.5 L/min FiO2 100%), at the corrected age of 16 weeks, she was readmitted to our emergency department with signs of respiratory distress and circulatory decompensation. Echocardiography demonstrated suprasystemic PH. Severe PH persisted despite initiated invasive mechanical ventilation, triple vasodilating therapy [iNO, macitentan, and continuous intravenous (IV) sildenafil], as well as levosimendan, milrinone, and norepinephrine for recompensation from cardiac shock. Thus, we started off-label oral selexipag therapy (oral IP receptor agonist) in the smallest patient reported so far (4 kg body weight). Subsequently, RV systolic pressure decreased to half-systemic, allowing successful weaning of iNO, norepinephrine, and milrinone, and extubation of the patient over 4 days. The infant was discharged 4 weeks after pediatric intensive care unit (PICU) admission in stable cardiorespiratory condition, with an oral, specific, triple antihypertensive PAH-targeted therapy using selexipag, macitentan, and sildenafil as well as oxygen therapy at low-flow (0.5 l/min) and spironolactone. The first cardiac catheterization at the age of 9 months under aforementioned triple PAH-targeted therapy revealed mild PH with 35% systemic PA pressure (mPAP/mSAP = 0.35) and isolated pulmonary vein stenosis. A transthoracic biopsy at the age of 12 months confirmed the diagnosis of BPD and further showed pulmonary interstitial glycogenosis and severe pulmonary capillary hemangiomatosis, without involvement of the pulmonary venules (chILD A2, A3, and B4 according to the Deutsch-Classification). The patient is currently in stable cardiorespiratory condition undergoing triple PH-targeted therapy including selexipag. This report highlights the potential benefits of the oral prostacyclin mimetic selexipag as an early add-on PH-targeted drug in chronic PH of infancy (cPHi).

## Introduction

Bronchopulmonary dysplasia (BPD) is a condition with impaired respiratory function of the newborn, that primarily affects preterm infants, defined as the need for respiratory support (high-flow-nasal cannula, continuous positive airway pressure, mechanical ventilation) even in the absence of supplemental oxygen at corrected age of 36 weeks (1). The imbalance between lung-injury and -repair processes in developing, still immature lungs, alveolar simplification, and the arrest of vascular growth are key determinants in the pathophysiology of BPD (2–5). Frequently, BPD is associated with pulmonary hypertension (PH) (6–8). Indeed, in the context of BPD, disturbance of alveolar diffusion, abnormal vascular remodeling as well as the rarefication of pulmonary vessels (growth-arrest) lead to an increasing pulmonary vascular resistance (PVR) with subsequent RV failure (6, 7). About 25% of infants with moderate to severe BPD develop secondary PH (Group 1.6/3.5 PH) (9). About 31–47% of preterm infants die within 2 years after diagnosis of BPD-associated PH (BPD-PH) (10, 11). In particular, extremely premature infants born at 23–25 weeks of gestation are at substantial risk for PH (12). Beyond the substantial mortality, BPD-PH is also associated with impaired body growth, neurodevelopmental disorders, high oxygen demand, feeding problems, and a higher hospitalization rate than isolated BPD (13).

Advanced pharmacotherapy for pediatric PH undergoes rapid development today. Currently, many drugs with different routes of administration and targeted pathways are used to reduce PVR and consequently pressure overload on the RV, mainly by pulmonary vasodilatation (and anti-inflammation, anti-proliferation): phosphodiesterase-5 (PDE5) inhibitors (sildenafil), endothelin-receptor-antagonists (ERA) (macitentan, bosentan), soluble guanylate cyclase (sGC) stimulators (riociguat), prostacyclin agonists/analogues/mimetics (treprostinil, iloprost, epoprostenol, selexipag) (8). The majority of aformentioned PAH-targeted drugs, including selexipag are currently used off-label in children with PH in expert centers (14). Selexipag is the first orally administered selective prostacyclin receptor (IP) agonist that has been shown to induce vasodilation and to inhibit pulmonary (peri)vascular inflammation and –proliferation. Although our recently published multicenter study on add-on selexipag treatment in severe pediatric PH (15) highlighted the safety and efficancy of the drug, its use in preterm infants with PH has not been investigated so far. The following case report focuses on the evaluation and discussion of selexipag treatment in a preterm infant with chronic PH of infancy (cPHi) (6) and demonstrates the diagnostic work up needed in complex BPD-PH.

## Case report

### Baseline patient characteristics

The affected female preterm infant was born at 25 3/7 weeks postmenstrual gestational age, weighing 430 g [small for gestational age (SGA)]. Preterm birth due to maternal eclampsia and pathologic cardiotocography (CTG), lead to delivery by primary cesarean section. The postnatal chest x-ray (CXR) in our neonatal intensive care unit (NICU) showed acute respiratory distress syndrome (ARDS) grade 2–3 that required endotracheal intubation and mechanical ventilation. On the second day of life, echocardiography (Echo) showed severe persistent pulmonary hypertension in the newborn (PPHN). Thus, we initiated inhaled nitric oxide (iNO), muscle relaxation and continuous intravenous sildenafil-infusion. Intravenous (IV) dexamethason was applied at the age of 3 weeks. Subsequently the infant could be extubated, followed by continuous positive airway pressure support (CPAP). A hemodynamically significant patent ductus arteriosus (PDA) (16) was initially treated with ibuprofen and indomethacin, but ultimately closed by surgical ligation at 7 weeks of postnatal age. The postoperative Echo revealed tricuspid regurgitation (TR) grade 1 with an estimated RV systolic pressure (RVSP) of 3/4 systemic pressure and qualitatively sufficient biventricular systolic function. At 36 weeks of corrected gestational age, the patient was under CPAP respiratory support with 15 L/min flow and FiO2 of 80%, thus severe BPD according to the classification of BPD severity [Brumbaugh (19)] was present. CPAP was weaned and exchanged against low-flow oxygen supplementation with 1 L/min 100% O2 at 16 weeks of life-age (corrected age of 3 weeks). At the corrected age of 10 and 14 weeks, transthoracic echocardiography showed no TR, moderate dilation of the right atrium (RA), and an estimated half systemic RVSP. We discharged the infant in the 20th week of postnatal life, at the corrected age of 7 weeks and a body weight of 3650 g (10th percentile). At the time of discharge, she had not received any specific PH-targeted medication besides low flow supplemental oxygen since the PDA ligation.

### Acute decompensation in PH-bronchopulmonary dysplasia

In the 24th week of life, 4 weeks after NICU discharge, the patient presented in decreased general condition with cardio-pulmonary decompensation at our emergency department, with tachypnea, dyspnea, paleness, and lethargy. Laboratory tests revealed global respiratory failure with hypoxia (SpO_2_ 80%) and hypercapnia (pCO_2_ 106 mmHg), acidosis (pH 7.09, lactate 7.2 mmol/L, base deficit -3.2 mmol/L), as well as greatly increased cTroponin T (149 ng/L) and NT-proBNP (> 35.000 ng/L). Mean systemic blood pressure was low [mean systemic arterial pressure (mSAP) 25 mmHg] and central venous pressure high, so that we assumed severe PH crisis. A few days before the acute decompensation, the patient presented at our emergency department due to drops in oxygen saturation down to 50% in the patient’s domestic monitoring system and a mild infection of the upper respiratory tract. However, the patient was not admitted to our hospital due to stable cardiorespiratory status and normal oxygen saturation at presentation. No echocardiography was performed at first presentation. After immediate admission to our pediatric intensive care unit (PICU), the first Echo showed TR grade I with estimated suprasystemic RVSP (RVSP 81 mmHg + right atrial v wave, with systolic SAP of 63 mmHg), moderate to severe systolic RV dysfunction, and severe RV hypertrophy [increased right ventricular anterior wall thickness (RVAW) of 6 mm]. After primary intraosseous vascular access and endotracheal intubation, mechanical ventilation with iNO and oxygen supplementation was initiated. After placement of a central venous catheter, continuous infusions of sildenafil, epinephrine, and milrinone were established.

**FIGURE 1:**
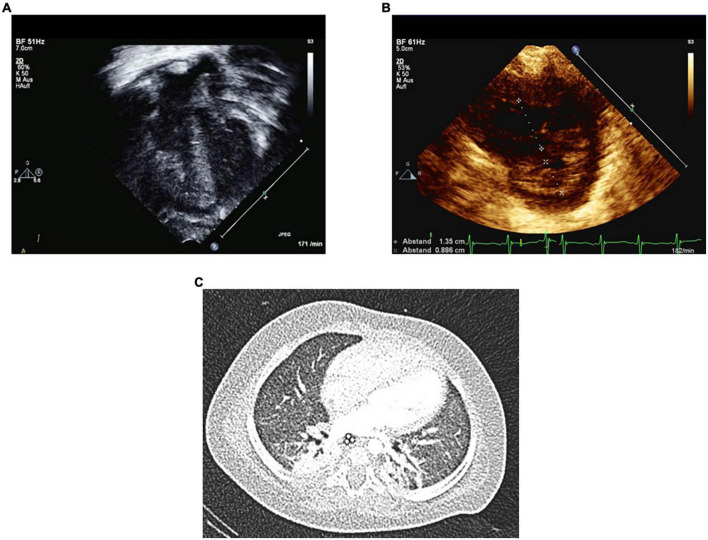
Echocardiography on day 2 and Chest CT on day 7 in PICU at the age of 6 months. Several Echo variables and ratios indicate severe PH on the second PICU day in acute cardiorespiratory decompensation. Panel **(A)** shows severe RV hypertrophy and RV dilation. Panel **(B)** shows increased RV/LV end-systolic diameter ratio of 1.5. Panel **(C)** shows chest CT with atelectasis of the right upper lobe and mild mosaic perfusion. No signs of cysts or bullae were identified.

On day 2 in PICU, transthoracic Echo still showed severe PH with an estimated RVSP of systemic arterial pressure level with a moderately dilated RV and moderate RV systolic dysfunction (Figures 1A,B). The pulmonary artery (PA) was much larger in caliber than the ascending aorta. The left ventricle (LV) appeared underfilled but had normal systolic function. Systemic arterial pressure was 76/46 (mean 61) mmHg. We consequently exchanged epinephrine for norepinephrine, started the ERA macitentan *per os*, transfused packed red blood cells (PRBC) (goal Hb 12–14 g/dl) and started levosimendan infusion the next day over a course of 24 h. Oral Spironolactone (5 mg twice daily) was added to the treatment regime on PICU day 1 (see Table 1 for all pertinent medications used during the PICU course). On day 4 in the PICU, 2 days after starting macitentan (dual PAH-targeted therapy), NTproBNP had decreased to 2.000 ng/L and Troponin T to 57 ng/L. Nevertheless, the patient remained dependent on intensive care including muscle relaxation, mechanical ventilation, iNO, additional oxygen, and continuous sildenafil infusion. Two attempts of weaning off both muscle relaxation and iNO ended up in severe, life threatening PH crisis, so that weaning mechanical ventilation was impossible.

**TABLE 1:** Medications used in PICU for infant (body weight 4.3 kg) with acute cardiorespiratory decompensation and PH crises.

*Oral PH-targeted medication*	Starting dose	Increase per dose	Final dose/day	PICU day	Comment
Sildenafil (four times daily)	1 mg per dose	1 mg	4 × 4 mg	1	Increase in CI. Decrease in PVR. Maintenance dose: 0.5–1 mg/kg/dose three (> 1 year old) or four times (< 1 year old) daily PO. In this case: transitioning from IV sildenafil.
Macitentan (once daily)	2 mg per dose	1 mg	1 × 4 mg	2	Increase in CI. Decrease in PVR. Not recommended in patients with moderate or severe hepatic impairment.
Selexipag (twice daily)	50 μg per dose	50–100 μg (see text)	2 × 400 μg	4	Prostacyclin IP receptor agonist. Reduction of morbidity/mortality event in adult PAH.
***Intravenous medication***					
Epinephrine	0.02 μg/kg/min	–	0.02 μg/kg/min	1–2	Positive inotrope. Increases myocardial oxygen consumption, tachycardia.
Norepinephrine	0.2 μg/kg/min	–	0.2 μg/kg/min	2–7	Increases SVR and PVR.
Sildenafil	3.1 mg/kg/day	–	3.1 mg/kg/day	2–9	Sildenafil 1–4 mg/kg/day, depending on systemic blood pressure.
Milrinone	0.7 μg/kg/min	–	0.7 μg/kg/min	2–8	Lowers PVR. Caution: systemic arterial hypotension.
Levosimendan	0.12 μg/kg/min	–	0.12 μg/kg/min	3	Lowers PVR. Caution: systemic arterial hypotension. Long half-life.
Morphine	0.18 mg/kg/h	–	0.18 mg/kg/h	2–9	Slowly weaned off during PICU stay.
Midazolam	0.09 mg/kg/h	–	0.09 mg/kg/h	2–9	Slowly weaned off during PICU stay.
*Cis*-atracurium	0.32 mg/kg/h	–	0.32 mg/kg/h	2–4	During mechanical ventilation.
Rocuronium	1 mg/kg/dose	–	1 mg/kg/dose	4–5	Single dosing. In this case: transitioning from *cis*-atracurium.
***Inhaled medication***					
Nitric oxide	20 ppm	–	20 ppm	2–6	Monitor Met-Hemoglobin. Selective fall in PVR. Rebound PH on weaning off iNO (risk can be reduced by concomitant use of sildenafil).

All medications that we administered in PICU with effects on hemodynamics, are listed. Total stay on PICU were 9 days. Comments are from the EPPVDN consensus statement on the treatment of pediatric pulmonary hypertension, please refer for detailed information (DOI: 10.1016/j.healun.2019.06.02).

### Add-on selexipag as pulmonary hypertension rescue therapy

Subsequently, on day 4 in the PICU, we initiated add-on off-label Selexipag treatment with a starting dose of 50 μg 0–0–1, increased to 50 μg twice daily (1–0–1) the next day and then gradually increased the dose by 50 μg daily (first 2 days), and then by 100 μg daily to a final dose of 400 μg/12 h. Technically, the 200 or 400 μg selexipag tablet was dissolved in 10 ml water and the according dose was administrated *via* the nasogastric tube. The cardiorespiratory condition then improved quickly: We stopped muscle relaxation (*cis*-atracurium) and started weaning iNO on PICU day 5, slowly weaned off iNO and norepinephrine at PICU day 6 and 7, and ultimately extubated the patient against high flow and weaned off milrinone on PICU day 8. Lastly, we were able to transfer the patient to the intermediate care unit (IMC) under tripple PH targeted therapy (sildenafil, macitentan, selexipag) 9 days after PICU admission (Table 1).

Further in the clinical course, the patient required intravenous broad-spectrum antibiotics (meropenem and vancomycin) over a span of 14 days, due to central venous line (CVL) associated sepsis, without the necessity for new intensive medical treatment. On day 33 after PICU admission (08/25/2020), discharge from the hospital was feasible at a body weight of 5 kg and tripple antihypertensive PAH-targeted medication (sildenafil 4 mg q 6 h; macitentan 4 mg once daily, selexipag 400 μg q 12 h) as well as low flow oxygen supplementation (0,5 L/min 100% O2).

### Diagnostic work up

#### Chest computed tomography

The patient underwent a chest-computed tomography (CT) already on day 9 in the PICU at the age of 6 months, in order to evaluate the pulmonary parenchyma and vessels (Figure 1C). CT images showed hypoplasia of lung parenchyma with alveolar simplification and diffuse mosaic perfusion, typically associated with extreme premature birth. No cysts or bullae were identified. Additionally, we found an atelectasis of the right upper lobe, which was bronchoscopically flushed out during the same inpatient stay.

#### Right-left-heart-catheterization

**FIGURE 2:**
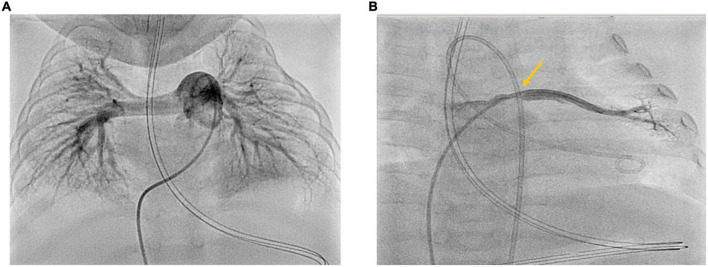
**(A,B)** Cardiac catheterization 3 months after PICU discharge at the age of 9 months. Right-Left heart catheterization 3 months after acute cardiorespiratory decompensation and initiation of triple oral PH-targeted medication (Sildenafil, Macitentan, Selexipag). Panel **(A)** shows angiography of the dilated proximal pulmonary artery: PVRi 4.1 WU•m^2^; mPAP 23 mmHg; mSAP 48 mmHg; mPAP/mSAP 0.48 (<half systemic PAP). Panel **(B)** shows angiography of the left upper pulmonary vein with the stenosis at entry to LA with LUPV pressure of 21–22 mmHg and dP mean LUPV to LA of 12 mmHg.

A total of 3 months after PICU discharge and initiation of triple combination antihypertensive PH-targeted drug therapy, we performed right-left heart catheterization (RLHC) (Figure 2) at the age of 9 months. RLHC is essential for an accurate diagnosis of PH (8, 17, 18) and for new findings regarding relevant primary or secondary pathologies. Pressures derived from RLHC were as follows (mmHg): mean right atrial pressure (mRAP): 6; RV systolic/diastolic pressure: 34/6; pulmonary arterial pressure (PAP) systolic/diastolic: 29/17; mean PAP (mPAP): 23; systemic arterial pressure (SAP) systolic/diastolic: 76/47; mean SAP (mSAP): 61; pulmonary artery wedge pressure (PAWP): 10–12. The mPAP/mSAP ratio was 0.38, i.e., below half systemic PA pressure, and strongly dropped compared to PICU admission 3 months earlier (supra systemic RVSP). Pulmonary vascular resistance (PVR: 4.1 WU•m^2^) was moderately elevated. PVR/SVR ratio was 0.26. Considering Cardiac Index (Qsi: 3.55 l/min/m^2^), the Qp/Qs ratio was 0.9. According to the 6th-World Symposium on PH (WSPH)–hemodynamic definition of PH (19), all criteria of isolated precapillary PH were fulfilled. Under the condition of 30 ppm iNO and 100% Oxygen, the acute vasoreactivity test (AVT) was negative as defined by the EPPVDN (i.e., neither a decrease of the mPAP nor of the PVR/SVR ratio by at least 20%) (8). Furthermore, by angiography, we could identify an isolated pulmonary vein stenosis (PVS) at the orifice of the left upper pulmonary vein (LUPV) in the left atrium (LA), with a LUPV pressure of 22 mmHg, mean LA pressure of 10 mmHg, and thus a pressure gradient dP mean LUPV to LA of 12 mmHg. The other three pulmonary veins were not obstructed, so we did not consider further intervention necessary at this stage.

#### Lung biopsy

**FIGURE 3:**
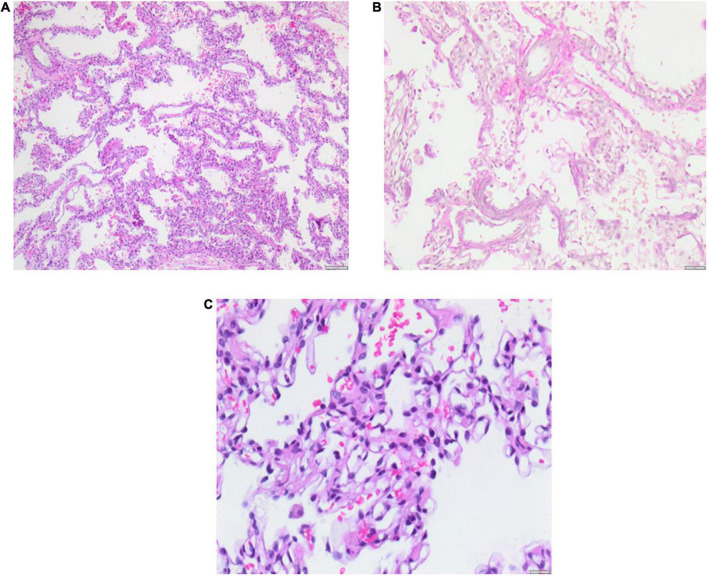
Lung biopsy 5 months after PICU discharge at the age of 11 months. Histology shows pulmonary capillary hemangiomatosis (PCH) without pulmonary venous involvement and mild pulmonary interstitial glycogenosis (PIG) with small foci. Severe bronchopulmonary dysplasia (BPD) is evident as alveolar simplification/hypoalveolarisation. Scale bar = 100 μm.

Lung biopsy is an invasive diagnostic method for the classification of diffuse parenchymal lung disease (DPLD) or so-called children’s interstitial lung disease (chILD). Before lung biopsy, and solely based on the medical history and non-invasive diagnostics, the patient was classified as group A2 (DPLD-Growth abnormalities deficient alveolarization) and group B4 (DPLD-related to lung vessels structural processes) according to the Deutsch-Classification (20, 21). We performed lung biopsy due to recurring severe respiratory infections, treated with multiple steroid pulses with moderate effects. Lung biopsy was performed by pediatric surgeons through a posterolateral mini-thoracotomy under general anesthesia without any complications, at the corrected age of 9 months. Histology confirmed BPD with hypoalveolarisation and an abnormal structure of the pulmonary vasculature with smooth muscle cell proliferation and altered extracellular matrix. A major histological finding was a variable pulmonary capillary hemangiomatosis (PCH), showing proliferating capillaries in row and grape-like formations around the alveolar septal tissue, that partly infiltrated alveolar septal tissue resulting in septal thickening. No participation or alteration of the pulmonary veins, particularly no obliterative fibrosis of the post-capillary venules was found. Small areas of the biopsy showed mild pulmonary interstitial glycogenosis (PIG) with cytoplasmic accumulation of glycogen in the interstitial cells (Figure 3).

#### Genetic testing

Gentic testing by means of whole exom sequencing (WES) revealed a heterozygous mutation in the Endoglin (ENG) gene (c.172_1728del; p.{lle575del}) in the infant and the mother as a variant of unknown significance (VUS). There was no other pathogenic mutation in the following potential disease-causing genes (PH- and ILD-Panel): ABCA3, CSF2RW, FLNA, FOXF1, SFTPB, SFTPC, TBX4, ACVRL1, BMPR2, CAV1, EIF2AK4, GDF, KCNA5, KCNK3, SMAD4, SMAD9, AGPAT2, ALMS1, BSCL2, FOS, PPARG.

#### Follow-up and outcomes

In the further course, several upper respiratory tract infections (URTI) (5–6×/year) lead to exacerbations of cardiorespiratory condition, that frequently required high flow oxygen and antibiotic treatment. The patient regularly responded well to systemic steroid pulse therapy in severe respiratory exacerbations (10–20 mg/kg/day methylprednisolon on 3 consecutive days).

Currently, feeding difficulties and slow growth are still present: The patient is still completely dependent on high calorie tube feeding through a percutaneous endoscopic gastrostomy (PEG) tube due to dysphagia and the inability of oral feeding. The patient is currently living with her family at home, supported by a pediatric nurse for outpatient care; she receives regular training for dysphagia and neurodevelopmental disorder. Follow-up visitis are conducted at a regular interval of 3 months in specialized pediatric cardiology and pulmonology clinics. Under a triple antihypertensive PAH-targeted medication (sildenafil, macitentan, selexipag), spironolactone, and low flow oxygen supplementation, the last Echo in April 2022 did not show any evidence for severe or higher grade PH. The patient had significant catch-up growth and is in very good general condition, with slow but steady motoric and mental development. At the current age of 2 years and 4 months, she crawls, pulls herself up on objects and is able to sing songs more and more understandably.

## Discussion

Generally, BPD is a heterogeneous condition with a variable degree of respiratory dysfunction, whose subtypes are hardly defined. BPD-PH due to pulmonary vascular disease (PVD) is characterized by alveolar simplification, impaired pulmonary angiogenesis, vascular inflammation, and obliterative pulmonary vascular remodeling, leading to respiratory failure and increasing PVR with consecutive RV-failure (6, 7). Within the classification of PH, BPD-PH is assigned to group 3: PH associated with lung disease (8). About 25–40% of infants affected by BPD, develop secondary chronic PH (22), a condition with a significantly higher mortality rate than in isolated BPD (10, 23).

We recently coined the term *“chronic PH of infancy (cPHi)”* (6) that applies to the infant described in this current case report. Besides developmental lung diseases including BPD, other conditions such as congenital cardiovascular disease with increased pulmonary blood flow, e.g., left-to-right shunting *via* a large PDA or VSD, frequently lead to elevated PVR and chronic PAH, if not treated in a timely fashion (approx. 4–8 months of age) (16). Depending on the degree of left to right shunting and related pulmonary hyperperfusion, patients are at risk of developing pulmonary edema, respiratory failure; they may also show abnormal remodeling of the pulmonary vascular bed with consecutive elevated PVR and chronic PH. Thus, we initiated pharmacotherapy with indometacin/ibuprofen for an early PDA closure without any success, so that the PDA ultimately had to be ligated surgically.

It is necessary to classify and unbundle BPD-PH and its sub-componentes through a diagnostic workup, whenever the patient is in stable cardiorespiratory condition. An accurate diagnostic workup, that may or may not include invasive lung biopsy, is essential for diagnosis. The histological findings are very relevant to understand primary or secondary pathologies in order to develop a sufficient treatment strategy. Lung biopsy represents the state of the art for diagnosis and classification of diffuse parenchymal lung disease (DPLD) or so called children’s interstitial lung disease (chILD) in pediatric pulmonology (26). In our case, invasive examinations such as lung biopsy and RLHC were performed under general anesthesia once the patient was in stable cardiorespiratory condition. Histology (Figure 3) revealed crucial findings for accurate classification of DPLD, so DPLD/chILD was classified as (1) PCH with variable expression (group B4: DLPD-related to lung vessel structural processes), (2) mild small-hearted PIG (group A3: DPLD-Infant conditions of undefined etiology), and (3) severe BPD with hypoalveolarization (group A2: DPLD-Grwoth abnormalitites/alveolarization abnormalities). The main histological finding was PCH with proliferating capillaries, that partly infiltrated alveolar septal tissue.

Pulmonary capillary hemangiomatosis with or without obstruction of pulmonary venules was first described in 1978 as a pulmonary vascular pathology (27). The infiltration of the excessively proliferating alveolar capillaries in alveolar septal tissue, bronchioles, and interstitial lung parenchyma plays an important role in the pathophysiology of PCH (28). The exact etiology of PCH is still not completely understood. Neoplastic processes and/or proliferation as a response to hypoxia are part of the assumed pathologic mechanisms (29). Currently, there is no curative therapeutic approach to PCH, rather, the disease has so far been treated with systemic steroids and vasodilative PH-targeted medication in case of PH. However, there is no standardized treatment strategy in children and infants with PCH, particularly due to the lack of published data on that topic. Fortunately, there was no pulmonary venous involvement in our patient in terms of a pulmonary veno-occlusive disease (PVOD). PVOD is characterized by obliteration of the small pulmonary venules through fibrotic thickening of the venous intima and irregular capillary proliferation (30), that lead to a progressive increase of PVR, PH and consecutive RV failure with high a mortality in patients with PVOD (31).

The patient of this report is currently treated with triple vasodilative and antiproliferative PH-targeted drugs. She also had intermittent systemic steroid pulses during exacerbations, which regularly and successfully led to a good cardiorespiratory condition. As of october 2022, no steroid pulses have been necessary for > 12 months, so that we can conclude that the underlying ChILD has substantially improved.

Generally, most antihypertensive PH-targeted drugs (except sildenafil and bosentan) are applied off-label in children with PH (14). Approval of PH-targeted drugs is limited mainly due to a lack of studies on the use of these drugs, especially in younger children and infants. Besides Sildenafil as a PDE5 inhibitor and Bosentan as an ERA, there is no data for the use of the other PH-targeted drug classes and their representatives for their application in BPD-PH (7). As in our case, macitentan is the primary ERA at our center and is preferred over bosentan mainly due to its less liver toxicity than bosentan, and therefore does not require regular liver function testing (blood draws) every 4 weeks. Furthermore, macitentan does not lower plasma sildenafil levels, as had been shown for bosentan. A prospective clinical phase 2 study on the safety, tolerability, and pharmacokinetics of selexipag in children is currently underway (estimated completion date: December 2026, trial ID: NCT03492177). Our prospective multicenter study on selexipag therapy in pediatric PH from 2020 (15) (*n* = 15, age range: 0.6–16.8 years) covered the safety and efficacy of selexipag in pediatric PH. Over 50% of the patient cohort showed a significant improvement in risk stratification, hemodynamic variables and physical activity after a median of 8 months add-on selexipag therapy. However, there is still no published data on selexipag treatment in preterm infants with BPD-PH. We started selexipag to support weaning off muscle relaxation, iNO and mechanical ventilation on pre-existing multiple antihypertensive PH-targeted drugs and circulatory support regimens. A decision was made to try oral selexipag based on our published multicenter study (15) first; as we expected it was much easier to discharge the patient as oral selexipag would allow for patient extubation, which was actually the case. IV epoprostenol or treprostinil would have been an option in severe PPHN or precapillary PH; but because we have seen patients with PVOD/PCH deteriorating quickly on IV treprostinil, we did not consider it suitable for the initial treatment. Although the diagnosis of PVOD/PCH was not confirmed at the start of selexipag initiation, another PH-targeted permanent drug with intravenous/subcutaneous administration (e.g., treprostinil) would have probably extended the stay in PICU, whereby the implantation of an intravenous/subcutaneous trepostinil-pump in an infant weighing < 5 kg would be hardly feasible. Regarding the clinical course, the transition from PICU to ICM is much easier with an oral prostacyclin agonist (selexipag) than with IV treprostinil. Furthermore, we already had positive experiences regarding oral selexipag therapy in pediatric patients with severe PAH (17). In case of further dependence on mechanical ventilation, the ultima ratio would have been the listing for bilateral lung transplantion with an uncertain outcome considering prematurity, young age, and poor general condition (dystrophy). Fortunately, we already had experience in our center with the use and side effects of selexipag in children with severe PH (15). In the current case, add-on Selexipag caused a rapid decrease in PVR with subsequent reduction in RV pressure loading and consecutive feasibility of extubation in stable cardiorespiratory conditions. Importantly, drug treatment with selexipag in small infants with severe PAH should be applied only in experienced centers and particularly in slow dose increases. Especially side effects such as peripheral vasodilation and decrease in systemic resistance and perfusion may result in multiple organ damage and limit therapy success. Another caveat is that an obstruction in the pulmonary capillary-pulmonary venous part of the circulation can not be excluded easily based on a chest CT or the clinical course, so that acute pulmonary edema can be a severe adverse event when using add-on Selexipag in this vulnerable patient population. Ultimately, the use and dose escalation of selexipag should be performed under regular blood pressure measurements and clinical monitoring, particularly regarding intestinal and renal function.

## Conclusion

This first-in-infant report highlights the potential benefits of the orally available prostacyclin mimetic selexipag (IP receptor agonist) as an efficient add-on PH-targeted drug in infants with severe, chronic PH.

## Data availability statement

The original contributions presented in this study are included in the article/supplementary material, further inquiries can be directed to the corresponding author.

## Ethics statement

Ethical review and approval was not required for the study on human participants in accordance with the local legislation and institutional requirements. Written informed consent to participate in this study was provided by the patients’ legal guardian.

## Author contributions

HH collected data and wrote the first draft of the manuscript. GH initiated the report, collected data, and edited the manuscript. All authors read the manuscript, edited the manuscript for important intellectual content, approved the final manuscript as submitted, and agreed to be accountable for all aspects of the work.
